# Gender Differences in the Diagnosis of Dyslipidemia: ESCARVAL-GENERO

**DOI:** 10.3390/ijerph182312419

**Published:** 2021-11-25

**Authors:** Cristina Soriano-Maldonado, Adriana Lopez-Pineda, Domingo Orozco-Beltran, Jose A. Quesada, Jose L. Alfonso-Sanchez, Vicente Pallarés-Carratalá, Jorge Navarro-Perez, Vicente F. Gil-Guillen, Jose M. Martin-Moreno, Concepción Carratala-Munuera

**Affiliations:** 1 Clinical Medicine Department, Miguel Hernandez University, 03550 San Juan de Alicante, Spain; cristina.soriano01@goumh.umh.es (C.S.-M.); adriana.lopezp@umh.es (A.L.-P.); dorozco@umh.es (D.O.-B.); vgil@umh.es (V.F.G.-G.); maria.carratala@umh.es (C.C.-M.); 2 Department of Preventive Medicine and Public Health, School of Medicine, University of Valencia, 46010 Valencia, Spain; Jose.L.Alfonso@uv.es (J.L.A.-S.); jose.martin-moreno@uv.es (J.M.M.-M.); 3 Preventive Medicine Service, General University Hospital Consortium, 46014 Valencia, Spain; 4 Health Surveillance Unit, Castellon Mutual Insurance Union, 12004 Castellon, Spain; pallarev@uji.es; 5 Department of Medicine, Jaume I University, 12071 Castellon, Spain; 6 Biomedical Research Institute INCLIVA, Hospital Clinico Universitario de Valencia, University of Valencia, 46010 Valencia, Spain; navarro_jorge@gva.es; 7 Ciber of Epidemiology and Public Health (CIBERESP), Instituto de Salud Carlos III, 28029 Madrid, Spain

**Keywords:** diagnostic inertia, gender differences, dyslipidemia, primary health care

## Abstract

Evidence shows that objectives for detecting and controlling dyslipidemia are not being effectively met, and outcomes differ between men and women. This study aimed to assess gender-related differences in diagnostic inertia around dyslipidemia. This ambispective, epidemiological, cohort registry study included adults who presented to public primary health care centers in a Spanish region from 2008 to 2012, with dyslipidemia and without cardiovascular disease. Diagnostic inertia was defined as the registry of abnormal diagnostic parameters—but no diagnosis—on the person’s health record in a window of six months from inclusion. A total of 58,970 patients were included (53.7% women) with a mean age of 58.4 years in women and 57.9 years in men. The 6358 (20.1%) women and 4312 (15.8%) men presenting diagnostic inertia had a similar profile, although in women the magnitude of the association with younger age was larger. Hypertension showed a larger association with diagnostic inertia in women than in men (prevalence ratio 1.81 vs. 1.56). The overall prevalence of diagnostic inertia in dyslipidemia is high, especially in women. Both men and women have a higher risk of cardiovascular morbidity and mortality.

## 1. Introduction

Cardiovascular diseases (CVD) are still the leading cause of mortality, accounting for 31% of all deaths worldwide. Most CVD can be prevented by acting on modifiable risk factors [1]; however, the evidence shows that targets for detecting and controlling these risk factors have not been fully achieved. Dyslipidemia is one of the main cardiovascular risk factors. Although its prevalence exceeds 50% in Europe [2] (specifically, it ranges from 31% to 50% in Spain [3]), it is the least considered and treated risk factor, and despite modest gains, its control is still insufficient [4,5]. The recent IBERICAN study [5] shows that only 25.8% of patients with dyslipidemia are well controlled.

Even though CVD is the main cause of death in women [6,7], it is still perceived as a man’s disease [8,9]. Women and men generally share the same cardiovascular risk factors, but these have differential effects according to gender. For example, in women metabolic syndrome is the most important risk factor for developing ischemic heart disease at an unusually young age [10]; smoking is more likely to cause coronary ischemia in women than in men [11]; and the onset of hypertension and dyslipidemia is later in women, but also more poorly controlled [12,13].

Since the turn of the century, understanding has grown around the need to focus more on sex- and gender-related differences in the prevention, diagnosis, and treatment of CVD [14]. In 2007, the American Heart Association published evidence-based guidelines focused on the primary prevention of CVD in women, which were later updated in 2011 as effectiveness-based guidelines [15]. Despite the improvements that this guidance promoted, evidence indicates that healthcare delivery and outcomes still differ between women and men. Particularly worrisome are findings that women with a similar level of CVD risk as men are less likely to receive treatment or preventive recommendations [14,16]. Furthermore, women are less likely to receive treatment intensification or achieve the optimal treatment effect [17,18]. When these differences systematically lead to gender inequalities related to established roles and stereotypes, this can be a determinant of differences in health outcomes [19].

Broadly speaking, the poor control of dyslipidemia in both sexes may be related, on the one hand, to limitations in the predictive capacity of the SCORE scale to detect cardiovascular disease [20], and on the other hand, to clinical inertia. Phillips was the first to define this concept in 2001 [21] as “the failure of physicians to initiate or intensify treatment when it was indicated”. Subsequently, the term has been reformulated as therapeutic inertia. Some studies on this topic, such as the one published by Chou AF et al. [22] in 2007, report low control of low-density lipoprotein (LDL) cholesterol in all patients, but especially in women, suggesting a less intensive cholesterol treatment in women, that is, greater therapeutic inertia in this group. Gil-Guillén et al.’s [23] working group differentiated between “diagnostic inertia,” or the failure to initiate treatment, and “therapeutic inertia,” or the failure to intensify it. In a systematic review on the concept of therapeutic inertia in arterial hypertension in primary care [24], review authors recognized the new definition of diagnostic inertia for the first time. Clinical inertia is frequent in pathologies such as arterial hypertension [25] or dyslipidemia; in a 2014 cross-sectional study, investigators observed that 38% of all cholesterol alterations and 17.7% of alterations in high-density lipoprotein (HDL) cholesterol were not diagnosed [26]. Regarding the factors associated with clinical inertia, Meador et al. [27] found that younger or obese people may be at higher risk of having their hypertension remain undiagnosed. Studies exploring the clinical inertia for dyslipidemia are scarce, Palazon et al. [26] observed that type-2 diabetes, non-smoking, previous coronary heart disease, blood pressure values, and body mass index were factors associated with diagnostic inertia for dyslipidemia. There is a lack of research analyzing specifically the gender association with clinical inertia.

Until the second half of the 20th century, women were not included in experimental studies, so much of the current knowledge about the main diseases affecting population health comes from studies carried out exclusively in men, with their results also applied to women [28]. This gender bias in research and the scant consideration of sex-related differences in clinical trials undermine the certainty of the evidence produced and may have negative consequences for health. In 2015, Vázquez et al. [29] identified a triple gender bias in the health system, while Ruíz-Cantero MT et al. [30] highlighted the importance of analyzing diagnostic criteria and normal cutoff points from a gender perspective, especially for diseases associated principally with men. In 2018, Aggarwal et al. [31] concluded that risk factors for ischemic heart disease should be stratified by sex. Although recent research shows detrimental gender biases in terms of diagnostic delay and errors in women [32], to our knowledge no study has assessed differences in the application of diagnostic criteria for dyslipidemia between men and women.

Therefore, the objectives of this study were to assess the number of men versus women who meet the diagnostic criteria for dyslipidemia but have not been diagnosed or treated in the primary care setting; to describe the profile of the patients affected by clinical inertia; to determine whether diagnostic inertia is associated with higher cardiovascular risk, as measured by commonly used scales; and to compare diagnostic inertia by sex.

## 2. Materials and Methods

### 2.1. Study Design

This cross-sectional study is part of a research project whose protocol is published elsewhere [33].

### 2.2. Population Study

Patients from the ESCARVAL-RIESGO study cohort (Estudio Cardiometabólico Valenciano, in English Valencian Cardiometabolic Study) [34] were selected as the population for the study, which included men and women with cardiovascular risk factors but no CVD (coronary heart disease or cerebrovascular disease) and attended in normal primary care practice between 2008 and 2012. Baseline data were collected from the electronic medical record (EMR) for patients meeting the inclusion criteria. Eligible patients were men and women aged 30 years or older, with no history of CVD event on enrolment or within a six-month baseline window following inclusion, and who met at least one of the following conditions: (a) registered diagnosis of dyslipidemia according to the International Classification of Diseases, 9th revision (ICD-9); (b) under treatment with lipid-lowering drugs; or (c) had at least one blood test showing cholesterol levels above the limits established by clinical practice guidelines for primary care [35,36], that is, total cholesterol of at least 200 mg/dL or HDL cholesterol less than 45 mg/dL. Patients with inconsistent or incomplete data in their EMR were excluded.

### 2.3. Study Variables

The primary outcome variable was diagnostic inertia of dyslipidemia, defined when a patient presented at least one analytical result showing altered total or HDL cholesterol, as established by clinical guidelines, in the baseline window period of six months, and without any recorded diagnosis of or treatment for dyslipidemia.

The rest of the study variables were described in the protocol [33] and were included as long as data were available for more than 50% of the sample. Sociodemographic information collected included age (grouped in bands of 30 to 49 years, 50 to 59 years, 60 to 69 years, and 70 years or more) and sex. Clinical variables were body mass index (BMI: normal< 25 kg/m^2^; overweight 25.0–29.9 kg/m^2^; obese ≥ 30.0 kg/m^2^), systolic blood pressure (normal <140 mmHg or elevated ≥ 140 mmHg) and diastolic blood pressure (normal < 90 mmHg or elevated ≥ 90 mmHg); behavioral factors: tobacco use (no, yes, ex-smoker); and analytical indicators: HDL cholesterol (normal < 45 mg/dL or abnormal < 45 mg/dL), LDL cholesterol (normal < 130 mg/dL or elevated ≥ 130 mg/dL), triglycerides (normal ≤ 150 mg/dL or elevated > 150 mg/dL), total cholesterol (normal ≤ 200 mg/dL or elevated > 200 mg/dL). When no data were available for a given variable, they were categorized as missing. In addition, we collected data on comorbidities according to the ICD-9 codes for: hypertension, diabetes mellitus, atrial fibrillation, retinopathy, peripheral arterial disease, chronic kidney disease, kidney failure, proteinuria, left ventricular hypertrophy, heart failure, and metabolic syndrome. Finally, variables related to medication use were collected for antiplatelet agents, insulin, oral antidiabetic drugs, antithrombotics, antihypertensive treatment, and statins or other lipid-lowering drugs.

Patients’ cardiovascular risk was assessed by means of the usual risk scales in this population: SCORE, which measures the risk of cardiovascular mortality, and REGICOR, which measures the risk of morbidity and mortality. The risk was calculated for patients aged 40 to 64 years for SCORE, and for those aged 35 to 74 years for REGICOR, according to the applicability of these scales as defined by the authors and described by Conroy et al. [37] and Marrugat et al. [38] in 2003.

All variables were collected from the EMR, a single centralized registry for the entire Valencian Community. The validity of the laboratory data was guaranteed by the existence of an online laboratory, also accessible to the entire Valencian Community, whose results are systematically validated by the analyst of each reference hospital.

### 2.4. Statistical Analysis

The number and proportion of patients affected by diagnostic inertia were calculated for the overall study population and by sex. To assess the presence of diagnostic inertia according to qualitative variables, 2 × 2 tables were constructed, and groups were compared using the chi-squared test.

To analyze whether diagnostic inertia was associated with a greater risk of cardiovascular mortality (SCORE) and morbidity and mortality (REGICOR), mean risk scores were calculated in patients who presented diagnostic inertia in dyslipidemia using the Student’s t-test, or the Welch test in the absence of homoscedasticity. Prevalence ratios for inertia were estimated with their 95% confidence intervals (CIs) at each level of the explanatory variables using multivariable Poisson regression models with robust variance [39], stratifying by sex. Variables for inclusion in the model were selected according to a stepwise procedure based on the Akaike Information Criterion (AIC). For each model, we report the likelihood ratio test (LRT) goodness-of-fit test, the AIC value, and the area under the receiver operating curve (ROC). To avoid the multiplicity problem derived from the analysis of subgroups by sex (i.e., in order not to increase the overall probability of finding significant results by the mere fact of carrying out many analyzes on different variables obtained in the study sample), the type I error was corrected using the Bonferroni method to 0.025. Analyses were performed using the statistical program IBM SPSS Statistics for Windows, v. 26.0 (IBM Corporation, Armonk, NY, USA) and R software, v.4.0.2 (R Core Team, Vienna, Austria).

## 3. Results

Of the 89,244 total patients included in the ESCARVAL cohort, 58,970 patients met our selection criteria: 27,311 (46.3%) men and 31,659 (53.7%) women. The mean age of the sample was 57.9 years (standard deviation [SD] 12.3) in men and 58.4 years (SD 13.3) in women. Most (81.9%, *n* = 48,300) had been diagnosed with dyslipidemia or had been prescribed treatment for this pathology, while 18.1% (*n* = 10,670) had altered lipid levels and were neither diagnosed nor under treatment, indicating diagnostic inertia. A higher proportion of women presented this outcome (20.1%, *n* = 6358) than men (15.8%, *n* = 4312; *p* < 0.001).

Table 1 shows the prevalence of clinical and analytical variables for all included men and for those presenting diagnostic inertia. This outcome was associated with younger age, normal weight (19.9%), elevated LDL cholesterol (19.8%), non-smoking (17.3%), high systolic (12.4%) and diastolic (13.5%) blood pressure, normal HDL cholesterol (18.6%), and high total cholesterol (21.3%) (*p* < 0.001 for all comparisons). Table 2 shows the results in men according to comorbidities. Diagnostic inertia was more frequent in those with hypertension (18.3%), without heart failure (15.8%), and without the peripheral arterial disease (16%) (*p* < 0.025) as well as in those not being treated with antiplatelet therapies, insulin, oral antidiabetics, or antithrombotics (*p* < 0.001).

In women (Table 3), diagnostic inertia was associated with younger age, normal weight (25.9%), being a smoker (22.7%) or ex-smoker (21.7%), and missing parameters on the EMR for LDL cholesterol (24.2%), blood pressure (27.7%), HDL cholesterol (25.3%), total cholesterol (25.5%), and triglycerides (24.1%). Table 4 shows the prevalence of diagnostic inertia according to comorbidities. A higher risk for inertia was observed in women without heart failure (20.2%), without atrial fibrillation (20.1%), without diabetes mellitus (21.3%), without arterial hypertension (21.4%), and without retinopathies (20.1%) (*p <* 0.025). By treatment, diagnostic inertia was more frequent in women who were not receiving antiplatelet agents, insulin, oral antidiabetic drugs, antithrombotics, or lipid-lowering drugs (*p <* 0.001).

Table 5 shows the mean risk scores for cardiovascular mortality (SCORE) and morbidity and mortality (REGICOR). Both men and women presenting diagnostic inertia carried a higher cardiovascular risk than those without inertia, although this risk was higher in men than in women.

The prevalence ratios for diagnostic inertia according to sex are shown in Table 6. Men and women affected by diagnostic inertia have a similar profile, although in women the magnitude of the association with younger age was larger. In addition, missing measurements for blood pressure, HDL cholesterol, and total cholesterol were more closely associated with diagnostic inertia in women than in men. Regarding the pathologies, hypertension showed a larger association with diagnostic inertia in women than in men (prevalence ratio 1.81 vs. 1.56, respectively). Both models fit the data well and have good classificatory ability.

## 4. Discussion

In a primary care setting, 18% of adults who met the diagnostic criteria for dyslipidemia do not have a registered diagnosis nor have they been prescribed treatment. This proportion was significantly higher in women (20.1%) than in men (15.8%). Patients affected by diagnostic inertia were relatively young; had a normal weight; did not smoke; presented alterations in systolic blood pressure, HDL cholesterol, total cholesterol, LDL cholesterol or triglycerides, or had missing values on their EMR. This pattern differed slightly between women and men, with younger age and missing analytical values showing a higher-magnitude association with diagnostic inertia in women. On the other hand, men who presented diagnostic inertia had higher cardiovascular risk scores for morbidity and mortality compared to women. In both groups, there is a lack of assessment of subclinical disease (comorbidities) and this may promote clinical inertia and determine the course of cardiovascular diseases.

Regarding the factors associated with diagnostic inertia, a diagnosis of arterial hypertension and younger age (30–49 years) had a greater association with inertia in women than in men. These results are similar to those described by Palazón et al. [26] in 2014, who observed that being a woman, being middle-aged (45–59 years), and having hypertension were associated with diagnostic inertia in dyslipidemia. One notable difference between their study and ours is that we calculated the proportion of patients presenting diagnostic inertia on the basis of a population meeting diagnostic criteria for dyslipidemia, whereas Palazón et al. [26] used patients that did not have a diagnosis of dyslipidemia as the denominator.

Other studies have studied diagnostic inertia in hypertension, although we are not aware of any that have performed an analysis stratified by gender. Johnson et al. [40] found that young adults with diabetes, higher blood pressures, or a female provider had a faster diagnosis rate in a region of the USA. On the other hand, recently, Meador et al. [27] reported that young age and obesity were factors associated with diagnosis inertia in hypertension among patients from the USA. In 2016, Pallares et al. [41] observed a high prevalence of inertia in patients from a Spanish region, although unlike our results in dyslipidemia, theirs showed that inertia was associated with male sex and older age. On the other hand, in their 2010 study, Gil-Guillén et al. [23] observed a higher level of inertia in women with hypertension, which is consistent with our results. Furthermore, those authors observed an association between inertia and non-smoking.

In 2021, a study was conducted on therapeutic inertia in dyslipidemia and hypertension in patients with type 2 diabetes mellitus [42]. The authors observed a significant delay in initiating treatment for primary prevention in both cases, regardless of cardiovascular risk, and in all age groups. However, the analysis was not stratified by sex. Indeed, despite the existence of studies on diagnostic inertia in dyslipidemia and hypertension, there are hardly any published studies that analyze the risk of morbidity and mortality related to diagnostic inertia according to sex. Diagnostic inertia should not be attributed solely to error; it may also be due to the primary care physician’s more conservative attitude toward treatment. However, our results add to the evidence of gender inequalities in dyslipidemia management. A meta-analysis in 2016 that analyzed statins prescriptions showed that women were 24% less likely to be prescribed statins and 48% more likely to be prescribed an inappropriate dose [43]. Moreno-Arellano et al. reported similar results in 2018 [44].

Possible inequalities in women’s health derived from the sex-related differences detected in this study could cause gender inequalities (roles, behaviors, and identities established by society that are assigned to women and men) [45] if it is confirmed that the professional decisions regarding the same health problem are different between men and women [46]. These differences could be related to gender stereotypes, which refer to a set of imposed, strongly assumed, ideas about the characteristics, attitudes, and aptitudes of women and men. The higher prevalence of diagnostic inertia in dyslipidemia in women could represent an indirect form of gender-based discrimination. Furthermore, gender roles (behaviors accepted as feminine and/or masculine) can influence health professionals’ decision-making when diagnosing or initiating treatment [30,32,43,44,45,46]. To improve women’s cardiovascular health, it is essential to raise awareness of the unique aspects of dyslipidemia in women, both among professionals and in the population. Physicians’ attitudes and practice can be key determinants of women reaching their dyslipidemia control targets. It is important that health professionals include gender equity among their aims and consider the objectives of gender-based medicine in their clinical practice [47].

This study has some potential limitations, which we have tried to mitigate but that nevertheless may have influenced the results. First, the selection of medical records is not completely free of possible errors [48]. Given that the information source corresponds to an electronic record, there could be differences in the degree and level of data recording depending on each health professional who attended the included patients. To minimize this risk, before preparing the ESCARVAL-RIESGO cohort [34], medical professionals in the primary care setting were offered training courses for using the EMR information system and registration data. Secondly, it was not possible to calculate the cardiovascular risk for all included patients because their age did not always fall in the appropriate range for the risk scales or because some data were unavailable. On the other hand, we believe that the study presented is innovative, since it is the first to our knowledge to examine the association between diagnostic inertia in dyslipidemia and gender bias. In addition, the data come from a large sample of patients who attended routine clinical practice in primary care, providing reasonable external validity to the study.

## 5. Conclusions

The overall prevalence of diagnostic inertia in dyslipidemia is high, especially in women. The profile of the patient who did not have a diagnosis or treatment for dyslipidemia, despite meeting the diagnostic criteria, was: aged under 50 years; normal weight; a non-smoker; alterations or unregistered values for blood pressure, HDL cholesterol, total cholesterol, LDL cholesterol and triglycerides; and/or a diagnosis of hypertension. This pattern was slightly different between women and men. In both, patients with diagnostic inertia were at a higher risk of cardiovascular morbidity and mortality, and this risk was higher in men.

From the perspective of clinical implications, primary care physicians should be alert to abnormal analytical values in order to reduce diagnostic inertia in dyslipidemia, especially in women who are not being properly identified, thus avoiding possible health inequalities derived from diagnostic inertia. The information provided by this study could be essential to improve clinical practice in the field of primary care, both in medicine and in nursing, helping to reduce the gender biases that are still prevalent in health care. However, further research is needed to explore the reason for the conservative attitude of primary care physicians in these types of patients.

Future studies should address the causes of the gender difference in the prevalence of diagnostic inertia and if this fact also occurs in other pathologies, such as hypertension or diabetes. Furthermore, longitudinal studies are necessary to verify that diagnostic inertia is associated with higher morbidity and mortality.

## Figures and Tables

**Table 1:** Prevalence of diagnostic inertia in men, according to physical and analytical variables.

		Total Men Meeting Diagnostic Criteria for Dyslipidemia		Diagnosis or Treatment for Dyslipidemia		Diagnostic Inertia		
		*n*	%	*n*	%	*n*	%	*p* Value
**Age, years**	30–49	7462	27.3%	6099	81.7%	1363	18.3%	<0.001
	50–59	6963	25.5%	5924	85.1%	1039	14.9%	
	60–69	7689	28.2%	6583	85.6%	1106	14.4%	
	≥ 70	5197	19.0%	4393	84.5%	804	15.5%	
**Body mass index ^a^**	Normal	2976	10.9%	2383	80.1%	593	19.9%	<0.001
	Overweight	10,309	37.7%	8720	84.6%	1589	15.4%	
	Obese	8723	31.9%	7383	84.6%	1340	15.4%	
	Missing	5303	19.4%	4513	85.1%	790	14.9%	
**Tobacco use**	No	9044	33.1%	7478	82.7%	1566	17.3%	<0.001
	Yes	9391	34.4%	7905	84.2%	1486	15.8%	
	Ex-smoker	8876	32.5%	7616	85.8%	1260	14.2%	
**Diastolic blood pressure ^b^**	Normal	12,547	45.9%	10,857	86.5%	1690	13.5%	<0.001
	Elevated	3959	14.5%	3000	75.8%	959	24.2%	
	Missing	10,805	39.6%	9142	84.6%	1663	15.4%	
**Systolic blood pressure ^c^**	Normal	8427	30.9%	7383	87.6%	1044	12.4%	<0.001
	Elevated	8106	29.7%	6499	80.2%	1607	19.8%	
	Missing	10,778	39.5%	9117	84.6%	1661	15.4%	
**HDL cholesterol ^d^**	Normal	8510	31.2%	6924	81.4%	1586	18.6%	<0.001
	Elevated	7579	27.8%	6390	84.3%	1189	15.7%	
	Missing	11,222	41.1%	9685	86.3%	1537	13.7%	
**Total cholesterol ^e^**	Normal	5213	19.1%	4738	90.9%	475	9.1%	<0.001
	Elevated	11,780	43.1%	9274	78.7%	2506	21.3%	
	Missing	10,318	37.8%	8987	87.1%	1331	12.9%	
**Triglycerides ^f^**	Normal	8078	29.6%	6296	77.9%	1782	22.1%	<0.001
		Elevated	7336	26.9%	6383	87.0%	953	13.0%	
		Missing	11,897	43.6%	10,320	86.7%	1577	13.3%	
**LDL cholesterol ^g^**	Normal	5834	21.4%	5003	85.8%	831	14.2%	<0.001
	Abnormal	8778	32.1%	7043	80.2%	1735	19.8%	
	Missing	12,699	46.5%	10,953	86.3%	1746	13.7%	

**Bold:***p* < 0.025. ^a^ Normal < 25 kg/m^2^; overweight 25.0–29.9 kg/m^2^; obese ≥ 30.0 kg/m^2^. ^b^ Normal < 90 mmHg, elevated ≥ 90 mmHg. ^c^ Normal < 140 mmHg, elevated ≥ 140 mmHg. ^d^ HDL: high-density lipoprotein, normal > 45 mg/dL, abnormal ≤ 45 mg/dL. ^e^ Normal ≤ 200 mg/dL, elevated > 200 mg/dL. ^f^ Normal ≤ 150 mg/dL, elevated > 150 mg/dL. ^g^ LDL: low-density lipoprotein; normal < 130 mg/dL, elevated ≥ 130 mg/dL.

**Table 2:** Prevalence of diagnostic inertia in men according to comorbidities and treatments.

		Total Men Meeting Diagnostic Criteria for Dyslipidemia		Diagnosis or Treatment for Dyslipidemia		Diagnostic Inertia		*p* Value
		*n*	%	*n*	%	*n*	%	
**Comorbidities**								
Heart failure	No	27,072	99.1%	22,783	84.2%	4289	15.8%	**0.009**
	Yes	239	0.9%	216	90.4%	23	9.6%	
Proteinuria	No	27,159	99.4%	22,880	84.2%	4279	15.8%	**0.045**
	Yes	152	0.6%	119	78.3%	33	21.7%	
Peripheral arterial disease	No	26,788	98.1%	22,507	84.0%	4281	16.0%	**<0.001**
	Yes	523	1.9%	492	94.1%	31	5.9%	
Atrial fibrillation	No	27,173	99.5%	22,879	84.2%	4294	15.8%	0.38
	Yes	138	0.5%	120	87.0%	18	13.0%	
Diabetes mellitus	No	19,954	73.1%	16,774	84.1%	3180	15.9%	0.27
	Yes	7357	26.9%	6225	84.6%	1132	15.4%	
Hypertension	No	14,285	52.3%	12,355	86.5%	1930	13.5%	**<0.001**
	Yes	13,026	47.7%	10,644	81.7%	2382	18.3%	
Renal failure	No	27,299	100.0%	22988	84.2%	4311	15.8%	-
	Yes	12	0.0%	11	91.7%	1	8.3%	
Left ventricular hypertrophy	No	27,307	100.0%	22,996	84.2%	4311	15.8%	-
	Yes	4	0.0%	3	75.0%	1	25.0%	
Chronic kidney disease	No	27,128	99.3%	22,844	84.2%	4284	15.8%	0.86
	Yes	183	0.7%	155	84.7%	28	15.3%	
Retinopathy	No	27,210	99.6%	22,910	84.2%	4300	15.8%	0.28
	Yes	101	0.4%	89	88.1%	12	11.9%	
Metabolic syndrome	No	27,215	99.7%	22,913	84.2%	4302	15.8%	0.053
	Yes	94	0.3%	86	91.5%	8	8.5%	
**Treatments**								
Antiplatelets	No	24,196	88.6%	20,145	83.3%	4051	16.7%	**<0.001**
	Yes	3115	11.4%	2854	91.6%	261	8.4%	
Insulin	No	26,815	98.2%	22,546	84.1%	4269	15.9%	**<0.001**
	Yes	496	1.8%	453	91.3%	43	8.7%	
Oral antidiabetics	No	24,117	88.3%	20,190	83.7%	3927	16.3%	**<0.001**
	Yes	3194	11.7%	2809	87.9%	385	12.1%	
Antithrombotics	No	24,465	89.6%	20,278	82.9%	4187	17.1%	**<0.001**
	Yes	2846	10.4%	2721	95.6%	125	4.4%	
Statins/lipid-lowering drugs	No	20,029	73.3%	16,845	84.1%	3184	15.9%	0.42
	Yes	7282	26.7%	6154	84.5%	1128	15.5%	

**Bold:***p* < 0.025.

**Table 3:** Prevalence of diagnostic inertia in men, according to physical and analytical variables.

		Total Women Meeting Diagnostic Criteria for Dyslipidemia		Diagnosis or Treatment for Dyslipidemia		Diagnostic Inertia		*p* Value
		*n*	%	*n*	%	*n*	%	
**Age, years**	30–49	8206	25.9%	5285	64.4%	2921	35.6%	**<0.001**
	50–59	7906	25.0%	6596	83.4%	1310	16.6%	
	60–69	8411	26.6%	7260	86.3%	1151	13.7%	
	≥70	7136	22.5%	6160	86.3%	976	13.7%	
**Body mass index ^a^**	Normal	5831	18.4%	4318	74.1%	1513	25.9%	**<0.001**
	Overweight	9554	30.2%	7850	82.2%	1704	17.8%	
	Obese	10,088	31.9%	8337	82.6%	1751	17.4%	
	Missing	6186	19.5%	4796	77.5%	1390	22.5%	
**Tobacco use**	No	22,259	70.3%	18,007	80.9%	4252	19.1%	**<0.001**
	Yes	6682	21.1%	5165	77.3%	1517	22.7%	
	Ex-smoker	2718	8.6%	2129	78.3%	589	21.7%	
**Diastolic blood pressure ^b^**	Normal	15,380	48.6%	13357	86.8%	2023	13.2%	**<0.001**
	Elevated	3425	10.8%	2650	77.4%	775	22.6%	
	Missing	12,854	40.6%	9294	72.3%	3560	27.7%	
**Systolic blood pressure ^c^**	Normal	10,629	33.6%	9313	87.6%	1316	12.4%	**<0.001**
	Elevated	8163	25.8%	6685	81.9%	1478	18.1%	
	Missing	12,867	40.6%	9303	72.3%	3564	27.7%	
**HDL cholesterol ^d^**	Normal	14,723	46.5%	12359	83.9%	2364	16.1%	**<0.001**
	Elevated	3230	10.2%	2709	83.9%	521	16.1%	
	Missing	13,706	43.3%	10,233	74.7%	3473	25.3%	
**Total cholesterol ^e^**	Normal	4383	13.8%	4017	91.6%	366	8.4%	**<0.001**
	Elevated	14,485	45.8%	11,754	81.1%	2731	18.9%	
	Missing	12,791	40.4%	9530	74.5%	3261	25.5%	
**Triglycerides ^f^**	Normal	11,870	37.5%	9720	81.9%	2150	18.1%	**<0.001**
	Elevated	5087	16.1%	4427	87.0%	660	13.0%	
	Missing	14,702	46.4%	11,154	75.9%	3548	24.1%	
**LDL cholesterol ^g^**	Normal	6002	19.0%	5036	83.9%	966	16.1%	**<0.001**
	Abnormal	10,534	33.3%	8796	83.5%	1738	16.5%	
	Missing	15,123	47.8%	11,469	75.8%	3654	24.2%	

**Bold:***p* < 0.025. ^a^ Normal < 25 kg/m^2^; overweight 25.0–29.9 kg/m^2^; obese ≥ 30.0 kg/m^2^. ^b^ Normal < 90 mmHg, elevated ≥ 90 mmHg. ^c^ Normal < 140 mmHg, elevated ≥ 140 mmHg. ^d^ HDL: high-density lipoprotein, normal > 45 mg/dL, abnormal ≤ 45 mg/dL. ^e^ Normal ≤ 200 mg/dL, elevated > 200 mg/dL. ^f^ Normal ≤ 150 mg/dL, elevated > 150 mg/dL. ^g^ LDL: low-density lipoprotein; normal < 130 mg/dL, elevated ≥ 130 mg/dL.

**Table 4:** Prevalence of diagnostic inertia in women according to comorbidities and treatments.

		Total Women Meeting Diagnostic Criteria for Dyslipidemia		Diagnosis or Treatment for Dyslipidemia		Diagnostic Inertia		
		*n*	%	*n*	%	*n*	%	*p* Value
**Comorbidities**								
Heart failure	No	31,337	99.0%	25,020	79.8%	6317	20.2%	**0.001**
	Yes	322	1.0%	281	87.3%	41	12.7%	
Proteinuria	No	31,551	99.7%	25,211	79.9%	6340	20.1%	0.38
	Yes	108	0.3%	90	83.3%	18	16.7%	
Peripheral arterial disease	No	31,513	99.5%	25,161	79.8%	6352	20.2%	**<0.001**
	Yes	146	0.5%	140	95.9%	6	4.1%	
Atrial fibrillation	No	31,553	99.7%	25,206	79.9%	6347	20.1%	**0.012**
	Yes	106	0.3%	95	89.6%	11	10.4%	
Diabetes mellitus	No	25,634	81.0%	20,169	78.7%	5465	21.3%	**<0.001**
	Yes	6025	19.0%	5132	85.2%	893	14.8%	
Hypertension	No	17,032	53.8%	13389	78.6%	3643	21.4%	**<0.001**
	Yes	14,627	46.2%	11,912	81.4%	2715	18.6%	
Renal failure	No	31,649	100.0%	25,292	79.9%	6357	20.1%	-
	Yes	10	0.0%	9	90.0%	1	10.0%	
Left ventricular hypertrophy	No	31,655	100.0%	25,298	79.9%	6357	20.1%	-
	Yes	4	0.0%	3	75.0%	1	25.0%	
Chronic kidney disease	No	31,541	99.6%	25,202	79.9%	6339	20.1%	0.28
	Yes	118	0.4%	99	83.9%	19	16.1%	
Retinopathy	No	31,561	99.7%	25,212	79.9%	6349	20.1%	0.007
	Yes	98	0.3%	89	90.8%	9	9.2%	
Metabolic syndrome	No	31,615	99.9%	25,266	79.9%	6349	20.1%	-
	Yes	43	0.1%	35	81.4%	8	18.6%	
**Treatments**								
Antiplatelets	No	26,478	83.6%	20,649	78.0%	5829	22.0%	**<0.001**
	Yes	5181	16.4%	4652	89.8%	529	10.2%	
Insulin	No	31,111	98.3%	24,803	79.7%	6308	20.3%	**<0.001**
	Yes	548	1.7%	498	90.9%	50	9.1%	
Oral antidiabetics	No	29,055	91.8%	23,025	79.2%	6030	20.8%	**<0.001**
	Yes	2604	8.2%	2276	87.4%	328	12.6%	
Antithrombotics	No	29,795	94.1%	23,591	79.2%	6204	20.8%	**<0.001**
	Yes	1864	5.9%	1710	91.7%	154	8.3%	
Statins/lipid-lowering drugs	No	23,955	75.7%	18,804	78.5%	5151	21.5%	**<0.001**
	Yes	7704	24.3%	6497	84.3%	1207	15.7%	

**Bold:***p* < 0.025.

**Table 5:** SCORE and REGICOR cardiovascular risk scores, according to inertia and sex.

Risk Score			n	Mean Risk Score	SD	*p* Value
**SCORE**	Men	Diagnosis or treatment	5946	2.94	2.73	**<0.001**
		Diagnostic inertia	1510	3.28	2.76	
		Mean difference		0.34		
	Women	Diagnosis or treatment	6061	1.10	1.07	**0.011**
		Diagnostic inertia	1508	1.19	1.24	
		Mean difference		0.09		
**REGICOR**	Men	Diagnosis or treatment	8346	6.85	4.61	**<0.001**
		Diagnostic inertia	2100	7.53	4.67	
		Mean difference		0.68		
	Women	Diagnosis or treatment	8859	3.93	2.85	**<0.001**
		Diagnostic inertia	2126	4.30	2.87	
		Mean difference		0.37		

**Bold:***p* < 0.025. SD: standard deviation.

**Table 6:** Multivariable Poisson regression, prevalence ratios (PRs) for diagnostic inertia, by sex.

		Men			Women		
		PR	(95% CI)	*p* Value	PR	(95% CI)	*p* Value
**Age, years**	30–49	1			1		
	50–59	0.76	(0.70–0.82)	<0.001	0.46	(0.43–0.49)	<0.001
	60–69	0.74	(0.69–0.80)	<0.001	0.36	(0.34–0.39)	<0.001
	≥ 70	0.81	(0.74–0.88)	<0.001	0.36	(0.33–0.39)	<0.001
**Body mass index ^a^**	Normal	1			1		
	Overweight	0.79	(0.72–0.85)	<0.001	0.83	(0.79–0.88)	<0.001
	Obese	0.76	(0.70–0.83)	<0.001	0.83	(0.78–0.88)	<0.001
	Missing	0.76	(0.69–0.84)	<0.001	0.86	(0.81–0.91)	<0.001
**Tobacco use**	No	1			1		
	Yes	0.91	(0.86–0.98)	0.007	0.81	(0.77–0.86)	<0.001
	Ex-smoker	0.87	(0.81–0.93)	<0.001	0.88	(0.82–0.95)	0.001
**Systolic blood pressure ^b^**	Normal	1			1		
	Elevated	1.44	(1.34–1.55)	<0.001	1.51	(1.41–1.63)	<0.001
	Missing	1.48	(1.39–1.58)	<0.001	1.93	(1.83–2.03)	<0.001
**HDL cholesterol ^c^**	Normal	1			1		
	Elevated	1.16	(1.08–1.24)	<0.001	1.27	(1.15–1.39)	<0.001
	Missing	1.51	(1.32–1.74)	<0.001	1.60	(1.39–1.83)	<0.001
**Total cholesterol ^d^**	Normal	1			1		
	Elevated	2.87	(2.57–3.21)	<0.001	2.87	(2.56–3.22)	<0.001
	Missing	1.69	(1.44–1.99)	<0.001	2.60	(2.23–3.02)	<0.001
**Triglycerides ^e^**	Normal	1			1		
	Elevated	0.51	(0.47–0.55)	<0.001	0.64	(0.59–0.70)	<0.001
	Missing	0.60	(0.53–0.66)	<0.001	0.77	(0.70–0.86)	<0.001
**LDL cholesterol ^f^**	Normal	1			1		
	Abnormal	0.72	(0.66–0.78)	<0.001	0.60	(0.56–0.65)	<0.001
	Missing	0.69	(0.62–0.78)	<0.001	0.64	(0.57–0.72)	<0.001
**Comorbidities**	PAD	0.57	(0.41–0.80)	0.001	0.33	(0.15–0.72)	0.005
	Diabetes	1.19	(1.12–1.27)	<0.001	-		
	Hypertension	1.57	(1.48–1.66)	<0.001	1.76	(1.66–1.85)	<0.001
	Metabolic syndrome	0.51	(0.27–0.95)	0.035	-		
**Treatments**	Antiplatelets	0.61	(0.54–0.68)	<0.001	0.61	(0.56–0.66)	<0.001
	Oral antidiabetics	-			-		
	Antithrombotics	0.32	(0.27–0.38)	<0.001	0.61	(0.52–0.71)	<0.001
	Statins/lipid-lowering drugs	-			-		
	Insulin	-			0.74	(0.56–0.96)	0.025
N			27,309			31,659	
N with diagnostic inertia			4310			6358	
LRT (*p* value)			1485 (<0.001)			2710 (<0.001)	
AIC			23,099			30,467	
Area under the ROC (95% CI)			0.681 (0.672–0.689)			0.728 (0.721–0.735)	

AIC: Akaike information criterion; CI: confidence interval; LRT: likelihood ratio test; PAD: peripheral arterial disease. ^a^ Normal < 25 kg/m^2^; overweight 25.0–29.9 kg/m^2^; obese ≥ 30.0 kg/m^2^. ^b^ Normal < 140 mmHg, elevated ≥ 140 mmHg. ^c^ HDL: high-density lipoprotein, normal > 45 mg/dL, abnormal ≤ 45 mg/dL. ^d^ Normal ≤ 200 mg/dL, elevated > 200 mg/dL. ^e^ Normal ≤ 150 mg/dL, elevated > 150 mg/dL. ^f^ LDL: low-density lipoprotein; normal < 130 mg/dL, elevated ≥ 130 mg/dL.

## Data Availability

Data sharing is not applicable to this study protocol.

## References

[1] World Health Organization (2017). Cardiovascular Diseases. Fact Sheets. http://www.who.int/mediacentre/fact-sheets/fs317/es/.

[2] World Health Organization Global Health Observatory (GHO) data. http://www.who.int/gho/ncd/risk_factors/cholesterol_prevalence/en/.

[3] Cordero A., Fácila L. (2015). Situación actual de la dislipemia en España: La visión del cardiólogo. Rev. Esp. Cardiol. Supl..

[4] Reiner Ž., De Backer G., Fras Z., Kotseva K., Tokgözoglu L., Wood D., De Bacquer D., Euroaspire Investigators (2016). Lipid lowering drug therapy in patients with coronary heart disease from 24 European countries—Findings from the EUROASPIRE IV survey. Atherosclerosis.

[5] Sanjurjo S.C., Díaz M.Á.P., Caro J.L.L., Carratalá V.P., García A.B., Padial L.R., Rodríguez Á.D., García J.P., Martín J.V., Pérez R.V. (2017). Características basales y manejo clínico de los primeros 3.000 pacientes incluidos en el estudio IBERICAN (Identificación de la población española de riesgo cardiovascular y renal). Semergen.

[6] Roth G.A., Abate D., Abate K.H., Abay S.M., Abbafati C., Abbasi N., Abbastabar H., Abd-Allah F., Abdela J., Abdelalim A. (2018). Global, regional, and national age-sex-specific mortality for 282 causes of death in 195 countries and territories, 1980–2017: A systematic analysis for the Global Burden of Disease Study 2017. Lancet.

[7] Benjamin E.J., Muntner P., Alonso A., Bittencourt M.S., Callaway C.W., Carson A.P., Chamberlain A.M., Chang A.R., Cheng S., Das S.R. (2019). Heart Disease and Stroke Statistics—2019 Update: A Report From the American Heart Association. Circulation.

[8] Gender Matters: Heart Disease Risk in Women. https://www.health.harvard.edu/heart-health/gender-matters-heart-disease-risk-in-women.

[9] Catapano A.L., Graham I., De Backer G., Wiklund O., Chapman M.J., Drexel H., Hoes A.W., Jennings C.S., Landmesser U., Pedersen T.R. (2016). 2016 ESC/EAS Guidelines for the Management of Dyslipidaemias. Eur. Heart J..

[10] Pradhan A.D. (2014). Sex differences in the metabolic syndrome: Implications for cardiovascular health in women. Clin. Chem..

[11] Huxley R.R., Woodward M. (2011). Cigarette smoking as a risk factor for coronary heart disease in women compared with men: A systematic review and meta-analysis of prospective cohort studies. Lancet.

[12] Lloyd-Jones D.M., Evans J.C., Levy D. (2005). Hypertension in adults across the age spectrum: Current outcomes and control in the community. JAMA.

[13] Virani S.S., Woodard L.D., Ramsey D.J., Urech T.H., Akeroyd J.M., Shah T., Deswal A., Bozkurt B., Ballantyne C.M., Petersen L.A. (2015). Gender disparities in evidence-based statin therapy in patients with cardiovascular disease. Am. J. Cardiol..

[14] Garcia M., Mulvagh S.L., Merz C.N.B., Buring J.E., Manson J.E. (2016). Cardiovascular Disease in Women: Clinical Perspectives. Circ. Res..

[15] Von Mering G.O., Arant C.B., Wessel T.R., McGorray S.P., Bairey Merz C.N., Sharaf B.L., Smith K.M., Olson M.B., Johnson B.D., Sopko G. (2004). Abnormal coronary vasomotion as a prognostic indicator of cardiovascular events in women: Results rom the national heart, lung, and blood institute-sponsored women’s ischemia syndrome evaluation (wise). Circulation.

[16] Abuful A., Gidron Y., Henkin Y. (2005). Physicians’ attitudes toward preventive therapy for coronary artery disease: Is there a gender bias?. Clin. Cardiol..

[17] Gu Q., Burt V.L., Paulose-Ram R., Dillon C.F. (2008). Gender differences in hypertension treatment, drug utilization patterns, and blood pressure control among us adults with hypertension: Data from the national health and nutrition examination survey 1999–2004. Am. J. Hypertens..

[18] Chou A.F., Scholle S.H., Weisman C.S., Bierman A.S., Correa-de-Araujo R., Mosca L. (2007). Gender disparities in the quality of cardiovascular disease care in private managed care plans. Womens Health Issues.

[19] World Health Organitation Género y Salud. https://www.who.int/es/news-room/fact-sheets/detail/gender.

[20] Bertomeu-González V., Maldonado C.S., Bleda-Cano J., Carrascosa-Gonzalvo S., Navarro-Perez J., López-Pineda A., Carratalá-Munuera C., Guillén V.F.G., Quesada J.A., Brotons C. (2019). Predictive validity of the risk SCORE model in a Mediterranean population with dyslipidemia. Atherosclerosis.

[21] Phillips L.S., Branch W.T., Cook C.B., Doyle J.P., El-Kebbi I.M., Gallina D.L., Miller C.D., Ziemer D.C., Barnes C.S. (2001). Clinical inertia. Ann. Intern. Med..

[22] Chou A.F., Brown A.F., Jensen R.E., Shih S., Pawlson G., Scholle S.H. (2007). Gender and racial disparities in the management of diabetes mellitus among Medicare patients. Womens Health Issues.

[23] Gil-Guillén V., Orozco-Beltrán D., Pérez R.P., Alfonso J.L., Redón J., Pertusa-Martínez S., Navarro J., Cea-Calvo L., Quirce- Andrés F., Merino-Sánchez J. (2010). Clinical inertia in diagnosis and treatment of hypertension in primary care: Quantification and associated factors. Blood Press.

[24] Lebeau J.P., Cadwallader J.S., Aubin-Auger I., Mercier A., Pasquet T., Rusch E., Hendrickx K., Vermeire E. (2014). The concept and definition of therapeutic inertia in hypertension in primary care: A qualitative systematic review. BMC Fam. Pract..

[25] Wall H.K., Hannan J.A., Wright J.S. (2014). Patients with undiagnosed hypertension: Hiding in plain sight. JAMA.

[26] Palazón-Bru A., Gil-Guillén V.F., Orozco-Beltrán D., Pallarés-Carratalá V., Valls-Roca F., Sanchís-Domenech C., Martin-Moreno J.M., Redon J., Navarro-Perez J., Fernandez-Gimenez A. (2014). Is the physician’s behavior in dyslipidemia diagnosis in accordance with guidelines? Cross-sectional ESCARVAL study. PLoS ONE.

[27] Meador M., Lewis J.H., Bay R.C., Wall H.K., Jackson C. (2020). Who Are the Undiagnosed? Disparities in Hypertension Diagnoses in Vulnerable Populations. Fam. Community Health.

[28] Heidari S., Babor T., De Castro P., Tort S., Curno S. (2019). Equidad según sexo y género en la investigación: Justificación de las guías SACER y recomendaciones para su uso. Gac. Sanit..

[29] Vázquez-Santiago S., Garrido Peña F. (2016). El enfoque de género en las necesidades de atención sociosanitaria. Enferm. Clin..

[30] Ruiz-Cantero M., Blasco-Blasco M. (2020). Perspectiva de género en epidemiología clínica. Aprendiendo con el caso de las espondiloartritis. Gac. Sanit..

[31] Aggarwal N.R., Patel H.N., Mehta L.S., Sanghani R.M., Lundberg G.P., Lewis S.J., Mendelson M.A., Wood M.J., Volgman A.S., Mieres J.H. (2018). Sex Differences in Ischemic Heart Disease. Advances, Obstacles, and Next Steps. Circ. Cardiovasc. Qual. Outcomes.

[32] Ruiz-Cantero M.T., Blasco-Blasco M., Chilet-Rosell E.P.A. (2020). Sesgos de género en el esfuerzo terapéutico: De la investigación a la atención sanitaria. Farm. Hosp..

[33] Carratala-Munuera C., Lopez-Pineda A., Orozco-Beltran D., Quesada J.A., Alfonso-Sanchez J.L., Pallarés-Carratalá V., Soriano-Maldonado C., Navarro-Perez J., Gil-Guillen V.F., Martin-Moreno J.M. (2021). Gender Inequalities in Diagnostic Inertia around the Three Most Prevalent Cardiovascular Risk Studies: Protocol for a Population-Based Cohort Study. Int. J. Environ. Res. Public Health.

[34] Orozco-Beltran D., Gil-Guillen V.F., Redon J., Martin-Moreno J.M., Pallares-Carratala V., Navarro-Perez J., Valls-Roca F., Sanchis-Domenech C., Fernandez-Gimenez A., Perez-Navarro A. (2017). Lipid profile, cardiovascular disease and mortality in a Mediterranean high-risk population: The ESCARVAL-RISK study. PLoS ONE.

[35] Galán A.M., Cuixart C.B., Álvarez F.V., Pérez J.N., Lobos-Bejarano J.M., Sánchez-Pinilla R.O., Rioboó E.M., Banegas J.R.B., Orozco-Beltrán D., Gil Guillén V. (2012). Recomendaciones preventivas cardiovasculares. Atención Primaria.

[36] Orozco-Beltrán D., Cuixart C.B., Sánchez J.J.A., Banegas J.R.B., Cebrián-Cuenca A.M., Gil Guillen V.F., Rioboó E.M., Pérez J.N. (2020). Recomendaciones preventivas cardiovasculares. Actualización PAPPS 2020. Atención Primaria.

[37] Conroy R.M., Pyörälä K., Fitzgerald A.E., Sans S., Menotti A., De Backer G., De Bacquer D., Ducimetière P., Jousilahti P., Keil U. (2003). Estimation of ten-year risk of fatal cardiovascular disease in Europe: The SCORE project. Eur. Heart J..

[38] Marrugat J., Solanas P., D’Agostino R., Sullivan L., Ordovas J., Cordón F., Ramos R., Sala J., Masià R., Rohlfs I. (2003). Estimación del riesgo coronario en España mediante la ecuación de Framingham calibrada. Rev. Española Cardiol..

[39] Zou G. (2004). A Modified Poisson Regression Approach to Prospective Studies with Binary Data. Am. J. Epidemiol..

[40] Johnson H.M., Thorpe C.T., Bartels C.M., Schumacher J.R., Palta M., Pandhi N., Sheehy A.M., Smith M.A. (2014). Undiagnosed hypertension among young adults with regular primary care use. J. Hypertens..

[41] Pallares-Carratalá V., Bonig-Trigueros I., Palazón-Bru A., Lorenzo-Piqueres A., Valls-Roca F., Orozco-Beltrán D., Gil-Guil-len V.F., Steering Committee ESCARVAL Study (2016). Analysing the concept of diagnostic inertia in hypertension: A cross-sectional study. Int. J. Clin. Pract..

[42] Ling J.Z.J., Montvida O., Khunti K., Zhang A.L., Xue C.C., Paul S.K. (2021). Therapeutic inertia in the management of dyslipidaemia and hypertension in incident type 2 diabetes and the resulting risk factor burden: Real-world evidence from primary care. Diabetes Obes. Metab..

[43] Ballo P., Balzi D., Barchielli A., Turco L., Franconi F., Zuppiroli A. (2016). Gender differences in statin prescription rates, adequacy of dosing, and association of statin therapy with outcome after heart failure hospitalization: A retrospective analysis in a community setting. Eur. J. Clin. Pharmacol..

[44] Moreno-Arellano S., Delgado-de-Mendoza J., Santi-Cano M.J. (2018). Sex disparity persists in the prevention of cardiovascular disease in women on statin therapy compared to that in men. Nutr. Metab. Cardiovasc. Dis..

[45] (2012). What a Difference Sex and Gender Make: A Gender, Sex and Health Research Casebook.

[46] Ruiz Ruiz-Cantero María T., Verdú-Delgado M. (2004). Sesgo de género en el esfuerzo terapéutico. Gac. Sanit..

[47] Regitz-Zagrosek V., Seeland U. (2012). Sex and gender differences in clinical medicine. Handb. Exp. Pharmacol..

[48] Casey J.A., Schwartz B.S., Stewart W.F., Adler N.E. (2016). Using electronic healthrecords for population healthresearch: A review of methodsand applications. Annu. Rev. Public Health.

